# A numerical model of birch pollen emission and dispersion in the atmosphere. Description of the emission module

**DOI:** 10.1007/s00484-012-0532-z

**Published:** 2012-03-13

**Authors:** M. Sofiev, P. Siljamo, H. Ranta, T. Linkosalo, S. Jaeger, A. Rasmussen, A. Rantio-Lehtimaki, E. Severova, J. Kukkonen

**Affiliations:** 1 Finnish Meteorological Institute, Helsinki, Finland; 2 EVIRA, Turku, Finland; 3 University of Turku, Turku, Finland; 4 Finnish Forest Research Institute, Helsinki, Finland; 5 Medical University of Vienna, Vienna, Austria; 6 Danish Meteorological Institute, Copenhagen, Denmark; 7 Moscow State University, Moscow, Russia

**Keywords:** Birch pollen, Pollen emission, Pollen forecasting, Dispersion modelling

## Abstract

A birch pollen emission model is described and its main features are discussed. The development of the model is based on a double-threshold temperature sum model that describes the propagation of the flowering season and naturally links to the thermal time models to predict the onset and duration of flowering. For the flowering season, the emission model considers ambient humidity and precipitation rate, both of which suppress the pollen release, as well as wind speed and turbulence intensity, which promote it. These dependencies are qualitatively evaluated using the aerobiological observations. Reflecting the probabilistic character of the flowering of an individual tree in a population, the model introduces relaxation functions at the start and end of the season. The physical basis of the suggested birch pollen emission model is compared with another comprehensive emission module reported in literature. The emission model has been implemented in the SILAM dispersion modelling system, the results of which are evaluated in a companion paper.

## Introduction

Pollen grains and spores have been known to disperse over long distances since the middle of the previous century (Erdtman 1931, 1935; Gregory 1961); however, it attracted greater attention only comparatively recently. The long-range transport of pollen and spores has two evident consequences: (i) short-term (hourly or daily) changes in the pollen concentrations over receptor regions, which cannot be predicted using only local observations, and (ii) a large-scale redistribution of genetic material along the atmospheric pathways (Lindgren et al. 1995). Recently, substantial progress has been made in understanding the first phenomenon (Corden et al. 2002; Damialis and Gioulekas 2005; Hjelmroos 1992; Latalova et al. 2002; Mahura et al. 2007; Ranta and Satri 2007; Rantio-Lehtimaki 1994; Siljamo et al. 2008c; Skjøth et al. 2008; Sofiev et al. 2006a). It was shown that, although the features of each specific long-range transport episode vary widely, there may be a systematic pattern in the springtime pollen redistribution in Europe: prevailing transport directions, main source regions and regions mainly receiving pollen, etc. There were several attempts to reveal such a pattern via a multi-annual analysis (Damialis and Gioulekas 2005; Siljamo et al. 2008a, c; 2006; Skjøth et al. 2009; 2008; 2007; Smith et al. 2008; Sofiev et al. 2006a; Stach et al. 2007), and the amount of material and the number of analysed cases are growing. However, the overall picture for Europe (i.e., systematic surveys of the main transport directions, frequency of long-range transport events, characteristic dispersion distances, source-receptor matrices, etc.) is still to be drawn.

Different taxa exhibit varying potential to the long-range transport of their pollen. Arguably, one of the most important allergenic species in Europe is birch. It is a strong allergy-provoking plant that sensitises a substantial fraction of the population in nearly all parts of Europe. In Northern Europe, it is the most abundant allergenic pollen type with approximately 15% of the population being sensitized to its allergens (WHO 2003). The distribution of silver birch (*Betula pendula* Roth.) and downy birch (*B. pubescens* L.) covers a wide area extending from the mountain regions of Southern Europe to the northernmost Fennoscandia and through Siberia to the east coast of Asia (Atkinson 1992; OECD 2003).

The pollen long-range transport has one peculiarity. It has been known for decades that the bulk of pollen is deposited near the source plant (Raynor et al. 1970; Tampieri et al. 1977; Wright 1952, 1953). However, birches as well as the other species (*Alnus, Carpinus, Corylus, Ostrya, Fagus, Quercus, Castanea*) belonging to the order Fagales are wind-pollinated trees generating vast amounts of pollen to ensure a sufficient level of fertilization of female flowers over receptor regions. Their pollen grains are quite small and light to facilitate the transportation of a substantial fraction (up to 1% for some plumes) of the released material over thousands of kilometres if weather conditions are suitable (Sofiev et al. 2006b). Since the total number of the released pollen grains is high and the threshold levels for provoking symptoms in sensitized people are quite low, the long-range transported pollen may still have a substantial health impact (Viander and Koivikko 1978).

A key tool for analysing the pollen distribution in air is the atmospheric dispersion modelling. Many studies address the local distribution of pollen and seeds (Arritt et al. 2007; Aylor et al. 2006; Kuparinen et al. 2007), mainly addressing the distribution of the genetically modified species and the possibility of unwanted spread of these plants. At a larger scale, the integrated approaches based on dynamic models that cover the main parts of the pollen life cycle and its atmospheric transport are being developed in several countries.

The European-scale operational System for Integrated modeLling of Atmospheric coMposition (SILAM) presented in the present paper has been adapted to pollen computations by an international consortium within the scope of the POLLEN project. Its pollen source term has been accepted for the allergenic computations by the MACC, a European modelling consortium. Various versions of the model have been used for forecasts of pollen distribution in Europe starting from 2005 (Sofiev et al. 2006a). Additionally, the system has been applied to the reanalysis of flowering seasons starting from 1997 (Siljamo et al. 2008c; Veriankaitė et al. 2010).

A regional-to-local scale system was started within the ASTHMA project in Southern Europe. Together with SILAM, these systems formed a demonstration service in the ESA PROMOTE project (GMES service element) from where the SILAM based service continued in the EU-PASODOBLE downstream service. A system called COSMO-ART has been developed at the University of Karlsruhe (Helbig et al. 2004; Vogel et al. 2008) and is being refined by MeteoSwiss; the objective of the development is operational forecasting for Central and South Western Europe. Integrated model development is also going on in Denmark with the regional ENVIRO-HIRLAM system (Mahura et al. 2009) and local scale OML systems. Ongoing regional activities also take place in the US (for example, Efstathiou et al. 2011).

The goals of the present paper are: (i) to present a birch pollen emission model that is implemented in SILAM; (ii) to highlight the main driving processes and the uncertainties inherent in the model; (iii) to compare the new model with another pollen emission algorithm reported in literature. A companion paper by Siljamo et al., (IJB, 2012) quantifies the performance of the SILAM system with the present pollen emission module.

## Input information: dispersion and flowering models, phenological and aerobiological data

### Components of the SILAM dispersion model

As shown by Sofiev et al. (2006a), the main components of virtually any comprehensive chemistry transport model can be used to describe the pollen dispersion: advection with wind, mixing due to turbulence, gravitational settling (the main mechanism of pollen dry deposition), and scavenging with precipitation. The pollen model presented in this paper is constructed as a part of the SILAM modelling system (Sofiev et al. 2008). The model dynamic core includes both Lagrangian (Sofiev et al. 2006b) and Eulerian (Galperin 2000; Sofiev 2002) advection-diffusion formulations. The removal processes are described via dry and wet deposition. The dry deposition of pollen is described via gravitational settling, which for birch pollen results in the characteristic dry deposition velocity ~1.2 cm s^-1^ (Sofiev et al. 2006a). Advantages and problems of the dry deposition algorithms are discussed by (Kouznetsov and Sofiev 2012). The SILAM wet deposition parameterization (Horn et al. 1987; Jylha 1991; Smith and Clark 1989; Sofiev et al. 2006b) is based on direct observations performed for moderately hydrophobic aerosols. It distinguishes between sub- and in-cloud scavenging by both rain and snow. The particle size dependence of the impaction scavenging is taken into account by increasing the scavenging rate for super-micron particles in relation to their settling velocity. For pollen, already moderate rain (~1 mm) results in >50% of particles removed from the air.

More information on various dispersion models and features of their components can be found in the review of Kukkonen et al. (2012).

### Thermal time flowering model

The parameterization of flowering in the current emission model follows a principle of two thresholds for the temperature sum (Linkosalo et al. 2010), which assumes that the timing of birch flowering is mostly driven by accumulated ambient temperature during a certain time period. As shown by Linkosalo et al. (2010) as well as by a number of modelling works on birch phenology, the temperature sum threshold for both the start and end of the season is stable from year to year (Häkkinen et al. 1998; Hänninen 1990, 1995; Linkosalo et al. 2008).

According to Linkosalo et al. (2010), the cumulative fraction *R* of pollen released from the beginning of a year until time *t* is piecewise linear and proportional to the temperature sum *H* during the main flowering season.

1 $$ R(t) = \left[ {\matrix{{*{20}{c}} {0,} \hfill & {H(t) < {H_{{fs}}}} \hfill & {\left( {before\,\,the\,\,season} \right)} \hfill \\ {\frac{{H(t) - {H_{{fs}}}}}{{{H_{{fe}}} - {H_{{fs}}}}},} \hfill & {{H_{{fs}}} < H(t) < {H_{{fe}}}} \hfill & {\left( {season\,\,goes\,\,on} \right)} \hfill \\ {1,} \hfill & {H(t) > {H_{{fe}}}} \hfill & {\left( {season\,\,is\,\,over} \right)} \hfill \\ } } \right. $$

The temperature sum thresholds for the start and end of the season, *H*_*fs*_ and *H*_*fe*_, as well as the form of the function, *H(t)*, have to be identified from observational data.

### Input data

To identify the parameters in Eq. 1, a database of more than 15,000 records of dates of phenological phases across Europe has been collected (Siljamo et al. 2008b). From that database, the date of the leaf unfolding subset (the largest in the database) was taken for the present study.

Observational data on pollen concentrations in air were obtained from the European Aeroallergen Network (EAN, https://ean.polleninfo.eu/Ean/) which receives the data from around 35 countries and 300 sites all over Europe. For 2006 (the year highlighted in this paper), the set includes 5,787 daily data points from 213 stations.

The temperature time series for phenological and aerobiological stations were extracted from the meteorological archive of the European Re-Analysis (ERA-40, http:///www.ecmwf.int). The ERA-40 data cover the period from 1957 until 2001 with the six-hour time resolution of the analysis. ERA-40 combined the modelling capabilities and data from the observational networks via data assimilation, thus representing the best available knowledge on the state of the atmosphere, land and surface.

During the main season, the actual meteorological parameters are required for predicting the pollen release rate. These data were extracted from the ECMWF archive of the operational forecast. The time resolution of this dataset was 3 hours, whereas the spatial grid had a spacing of 0.25°.

## Pollen emission model

Following Sofiev et al. (2006ab), the output of the pollen emission module is described as a release flux of pollen grains *E(t,i,j)*: the number of grains emitted from 1 m^2^ of birch forest within 1 sec in a given model grid cell *(i,j)*. For dispersion computations, the final emission flux *E*_*mdl*_ *(t,i,j)* is obtained from *E(t,i,j)* by multiplication with the fraction of the birch forest *ϕ(i,j)* and the grid cell area *S(i,j)*:

2 $$ {E_{{mdl}}}(t,i,j) = E(t,i,j)\varphi (i,j)S(i,j) $$

The term *E(t,i,j)* has to be decomposed using the double-threshold model of Eq. 1:

3 $$ E(t,i,j) = \frac{{dR}}{{dt}}{N_{{total}}}\,p(t,i,j)\,F(meteo) $$

Here, *N*_*total*_ is the total number of pollen grains released from 1 m^2^ of birch forest during the whole season, *p(t,i,j)* is the probability of a tree in the given grid cell to flower at the specific time moment, and *F(meteo)* is the meteorology-dependent dynamic flux correction.

### Probabilistic description of the emission flux

As shown by Siljamo et al. (2008b), the irreducible uncertainties in the season timing are large and exceed the meteorological turnover time (~3 days of persistence of the weather pattern at synoptic scale in Europe). As a result, any deterministic model of the flowering season is inaccurate; errors in, e.g., start of the season, will be comparable with or exceeding 3 days. Since the transport conditions are dictated by the meteorological situation, a shift in pollen release of more than that meteorological turnover time would lead to incorrect release parameters and to a different dispersion pattern of the emitted pollens. A solution implemented in SILAM describes the flowering in probabilistic terms. The approach is based on assuming a certain probability for an individual tree to flower during a specific day:

4 $$ {p_f}(H/{H_{{fs}}},\,\,N/{N_{{total}}}) = {p_{{fs}}}(H/{H_{{fs}}}){p_{{fe}}}(N/{N_{{total}}}) $$

Here the functions *p*_*fs*_ and *p*_*fe*_ describe the probability for a single tree to start and end the flowering, respectively, and *N* is the cumulative pollen amount released since the start of the season.

For a grid cell, *p*_*fs*_ and *p*_*fe*_ characterise the fraction of trees flowering at each particular time moment. These fractions are less than unity near the season start and end. They do not affect the total number of pollen grains released during the whole season *N*_*total*_ but rather extend the flowering period; it takes longer to release the prescribed *N*_*total*_ pollens if some trees are not flowering at the beginning and at the end of the season.

#### Start of the season

Before the season, with *H* approaching *H*_*fs*_, *p*_*fs*_ determines the gradual start of the pollen release from the grid cell. The specific shape of the *p*_*fs*_ function is uncertain and hardly important. The only crucial parameter is the duration of the flowering spin-up, i.e. the transition range *δ*_*H*_ (in terms of the relative temperature sum) between mean daily emission intensities of, say, 5% and 95% in the grid cell (i.e., 5% and 95% of trees flowering inside the grid cell). This parameter is not measured, but can be transformed into an observable quantity as follows:

5 $$ {\delta_H} \approx {\delta_t}\frac{1}{{{H_{{fs}}}}}{\left. {\frac{{dH}}{{dt}}} \right|_{{H = {H_{{fs}}}}}} = \frac{{({T_{{fs}}} - {T_{{c - o}}})}}{{{H_{{fs}}}}}{\delta_t} $$

Here, *δ*_*t*_ is the time lag between the flowering intensities of 5% and 95% and *T*_*fs*_ is the temperature at the first flowering day. From the point of view of phenological observations, δ_t_ represents the uncertainty of the determination of the phenological phases. Such uncertainty has been quantified by Siljamo et al. (2008b).

For simplicity, *p*_*fs*_ is assumed to be piecewise linear with regard to the temperature sum:

6 $$ {p_{{fs}}}\left( {\frac{H}{{{H_{{fs}}}}}} \right) \equiv {p_{{fs}}}(x) = \left[ {\matrix{{*{20}{c}} {0,\,\,\,\,\,\,\,\,\,\,\,\,\,\,\,\,\,\,\,\,\,\,\,x < 1 - {\delta_H}\,\,\,\,\,\,\,\,\,\,\,\,\,\,\,\,} \\ {\frac{{x - 1 + {\delta_H}}}{{2\delta }},\,\,\,\,\,1 - {\delta_H} < x < 1 + {\delta_H}} \\ {1,\,\,\,\,\,\,\,\,\,\,\,\,\,\,\,\,\,\,\,\,\,\,\,\,\,x > 1 + {\delta_H}\,\,\,\,\,\,\,\,\,\,\,\,\,\,\,\,} \\ } } \right. $$

Here, *x = H/H*_*fs*_. According to Siljamo et al. (2008b), a *δ*_*H*_ of around 20% can be used as a rough estimate.

The application of the blurring function (Eq. 6) results in (i) a gradual start of pollen release already when the temperature sum is approaching the threshold but is still below it; and (ii) all of the trees in a grid cell getting involved in the process somewhat later than the threshold is passed.

#### End of the season

The end of the season is determined based on the “open pocket” principle; in other words, the emission continues until *N = N*_*total*_ (rather than the end-of-season heat sum, which is not assumed in SILAM).

The total amount of pollen developed in catkins, *N*_*total*_, is a very uncertain parameter which is at present estimated semi-manually using the data from the previous year and introduced into the model as a prescribed fixed map. Some regional studies show the possibility of predicting this parameter based on meteorological data from the previous year (Rasmussen 2002); however, the approach is yet to be extended to the European scale.

Following the same approach as that of the *p*_*fs*_ function, *p*_*fe*_ reads as:

7 $$ {p_{{fe}}}\left( {\frac{N}{{{N_{{total}}}}}} \right) = {p_{{fe}}}(R) = \left[ {\matrix{{*{20}{c}} {1,\,\,\,\,\,\,\,\,\,\,\,\,\,\,\,\,\,\,\,\,\,\,\,\,R < 1 - {\delta_N}\,\,\,\,\,\,\,\,\,\,\,\,\,\,\,\,} \\ {\frac{{1 + {\delta_N} - R}}{{2\delta }},\,\,\,\,\,1 - {\delta_N} < R < 1 + {\delta_N}} \\ {0,\,\,\,\,\,\,\,\,\,\,\,\,\,\,\,\,\,\,\,\,\,\,\,\,\,R > 1 + {\delta_N}\,\,\,\,\,\,\,\,\,\,\,\,\,\,\,\,} \\ } } \right. $$

A *δ*_*N*_ estimate of around 20% is used in the current SILAM setup.

### Start and propagation of the flowering season

To obtain *dR*/*dt*, we used the most common temperature sum formulation in the thermal time models, i.e. an integral of daily temperature *T* above a cut-off level *T*_*c-o*_ starting from moment *t*_*0*_:

8 $$ H(t) = \int\limits_{{{t_0}}}^t {(T(t) - {T_{{c - o}}}) * 1(T(t) - {T_{{c - o}}})dt} $$

Here, *1(x)* is the Heaviside function that equals 0 for *x* < 0 and 1 for *x* > 0.

Then, the relative release rate becomes a piecewise linear function of temperature:

9 $$ \frac{{dR(t)}}{{dt}} = \left[ {\matrix{{*{20}{c}} {0,\,\,\,\,\,\,\,\,\,\,\,\,\,\,\,\,\,\,\,\,\,\,\,\,\,\,\,\,\,\,\,\,\,\,\,\,\,\,\,\,\,\,\,\,\,\,\,\,\,\,\,\,\,\,\,\,\,\,\,\,\,\,\,\,\,\,\,\,\,\,\,\,\,\,\,\,\,\,\,\,\,\,\,\,H(t) < {H_{{fs}}} * (1 - {\delta_H})} \\ {\frac{{{{{dH(t)}} \left/ {{dt}} \right.}}}{{{H_{{fe}}} - {H_{{fs}}}}} = \frac{{(T(t) - {T_{{c - o}}}) * 1(T(t) - {T_{{c - o}}})}}{{\Delta H}},\,\,\,{H_{{fs}}} < H(t);\,\,R < 1\,\,\,\,\,\,} \\ {0,\,\,\,\,\,\,\,\,\,\,\,\,\,\,\,\,\,\,\,\,\,\,\,\,\,\,\,\,\,\,\,\,\,\,\,\,\,\,\,\,\,\,\,\,\,\,\,\,\,\,\,\,\,\,\,\,\,\,\,\,\,\,\,\,\,\,\,\,\,\,\,\,\,\,\,\,\,\,\,\,\,\,\,R > 1 * (1 + {\delta_N})\,\,\,\,\,\,\,\,\,\,} \\ } } \right. $$

Equation 9 requires three parameters: the starting temperature sum threshold *H*_*fs*_, the difference between the thresholds for the start and the end of pollen release *ΔH = H*_*fe*_ *−H*_*fs*_, and the cut-off temperature *T*_*c-o*_. Inclusion of *δ*_*H*_ and *δ*_*N*_ accounts for the probabilistic flowering description. A somewhat unorthodox introduction of the end of the season via *R* rather than *H*_*fe*_ allows for explicit consideration of the actual meteorology.

### Corrections dependent on short-term meteorological conditions

During the main season, three meteorology dependent correction functions are applied to the dynamic release rate *E(t,i,j)*: for wind speed, relative humidity and precipitation rate.

Precipitation and humidity related corrections are derived from known “prohibiting” thresholds totally suppressing the pollen release. Until these thresholds are reached, these variables do not affect the release (neither do they promote it). Near the threshold, the piecewise linearly decreasing transition function is as follows:

10 $$ {f_{{thr}}}(x,{x_{{low}}},{x_{{high}}}) = \left[ {\matrix{{*{20}{c}} {1,\,\,\,\,\,\,\,\,\,\,\,\,\,\,\,\,\,\,\,\,\,x \leqslant {x_{{low}}}} \\ {\frac{{{x_{{high}}} - x}}{{{x_{{high}}} - {x_{{low}}}}},\,\,\,\,\,\,\,\,\,{x_{{low}}} < x < {x_{{high}}}} \\ {0,\,\,\,\,\,\,\,\,\,\,\,\,\,\,\,\,\,\,\,\,\,\,x \geqslant {x_{{high}}}} \\ } } \right] $$

The lower and upper thresholds of relative humidity are taken as *q*_*low*_ = 50% and *q*_*high*_ = 80%.

For precipitation, *P*_*low*_ = 0. For the selection of *P*_*high*_, several considerations have to be taken into account. Any noticeable rain suppresses the release and scavenges out the emitted grains. The pollen release can also be stopped by high relative humidity associated with rain, which covers wider areas than the rain event itself. However, short-term convective rains cover an area much smaller than the grid cell (Morel and Senesi 2002). Such scattered precipitation still allows the trees to emit pollen from the dry parts of the grid cell area. Taking into account the above uncertainties, the estimate *P*_*high*_ = 0.5 mm hr^*-1*^ (the grid cell average rate) is taken as the threshold suppressing the pollen emission.

For the wind dependent correction, three phenomena need to be taken into account: (i) in the case of low wind but developed thermal convection, turbulence alone is sufficient to kick-start the release by generating sub-grid convective winds, (ii) stronger wind promotes the release by picking the pollen grains from open catkins; (iii) after reaching some level, further increase in wind strength does not affect the release rate which is then limited by the availability of ready-to-fly pollen grains in the catkins. These phenomena can be included in a single function as follows:

11 $$ {f_{{wind}}} = {f_{{stagnant}}} + {f_{{promote}}}\left[ {1 - \exp \left( { - \frac{{U + w*}}{{{U_{{satur}}}}}} \right)} \right] $$

Here, *U* is the wind speed, w* is convective velocity scale, *U*_*satur*_ is the saturation wind speed, and *(f*_*stagnant*_ *+ f*_*promote*_ *)* is the maximum “promotion” that wind can give to the release rate. In stagnant conditions, Eq. 11 suppresses the release by the *f*_*stagnant*_ factor.

In the current SILAM version, *U*_*satur*_ = 5 m sec^-1^, *f*_*stagnant*_ = 0.5 and *f*_*promote*_ = 1, which implies no impact at wind speed ~1 m s^-1^ and three-fold stronger release at very strong wind in comparison with calm conditions.

The resulting emission rate is a product of the above described specific terms:

12 $$ {E_{{model}}} = \left[ {\matrix{{*{20}{c}} {0:\,\,\,\,\,\,\,\,\,\,\,\,\,\,\,\,\,\,\,\,\,\,\,\,\,\,\,\,\,\,\,\,\,\,\,\,\,\,\,\,\,\,\,\,\,\,\,\,\,\,\,\,\,\,\,\,\,\,\,\,\,\,\,\,\,\,\,\,\,\,\,\,\,\,\,\,\,\,\,\,\,\,\,\,\,\,\,\,\,\,\,\,\,\,\,\,\,\,\,\,\,\,\,\,\,\,\,\,\,\,\,\,\,\,\,\,\,\,\,\,\,\,\,\,\,\,\,\,H < {H_{{fs}}} * (1 - {\delta_H})\,\,\,\,\,\,\,\,} \\ \begin{gathered} S(i,j)\,\,\varphi (i,j)\,{N_{{total}}}\frac{{T - {T_{{c - o}}}}}{{\Delta H}}\,\,{f_{{wind}}}(U,w*)\,\,{p_{{fs}}}\left( {\frac{H}{{{H_{{fs}}}}}} \right)\,\,\,{p_{{fe}}}(R)\,\, * \hfill \\ \,\,\,\,\,\,\,\,\,\,\,\,\,\,\,\,\,\,\,\,\,\,\,\,\,\,\,\,\,\,\,\,\,\,\,\,\,\,\,\,\,\,\,\,\,\,\,\,\,\,\,\,\,\,\,\,\,\,\,\,{f_{{thr}}}(q,{q_{{low}}},{q_{{high}}})\,\,{f_{{thr}}}(P,{P_{{low}}},{P_{{high}}}):\,\,\,\,H > {H_{{fs}}},R < 1 + {\delta_N} \hfill \\ \end{gathered} \\ {0:\,\,\,\,\,\,\,\,\,\,\,\,\,\,\,\,\,\,\,\,\,\,\,\,\,\,\,\,\,\,\,\,\,\,\,\,\,\,\,\,\,\,\,\,\,\,\,\,\,\,\,\,\,\,\,\,\,\,\,\,\,\,\,\,\,\,\,\,\,\,\,\,\,\,\,\,\,\,\,\,\,\,\,\,\,\,\,\,\,\,\,\,\,\,\,\,\,\,\,\,\,\,\,\,\,\,\,\,\,\,\,\,\,\,\,\,\,\,\,\,\,\,\,\,\,\,\,\,\,\,\,\,R > 1 + {\delta_N}\,\,\,\,\,\,\,\,\,\,\,\,\,\,\,\,\,} \\ } } \right] $$

One of the results of accounting for these corrections is that the heat sum at the end of the season is neither predictable nor important: it is not only temperature that determines the release during the main season. The *H*_*fe*_ is needed only to determine *ΔH*.

## Determination of parameters of the temperature sum model

The parameters needed for Eq. 12, namely, *H*_*fs*_ *, ΔH, T*_*c-o*_ *, t*_*0*_, were identified by fitting optimally the flowering start and end dates into the phenological and aerobiological observations. To overcome the problems related to the regional variations of these parameters, the European continent was split into 33 sub-regions which together cover its entire territory and have a limited but noticeable overlap with each other (~10% of their areas). The independent fittings were performed inside each region. The overlap between the regions resulted in partly overlapping sets of observations used by the fitting procedures in the neighbouring regions. This smoothed out the contrasts between the parameterizations in the neighbouring regions.

Since none of the above parameters is observed directly, the fitting variables were the dates of the phenological phases, such as the leaf unfolding date. These dates are the primary outputs of the phenological model Eqs. 8 and 9 and are also observed directly. The fitting then minimised the difference between the observed dates and the corresponding model predictions by varying the above parameters. To compute the temperature sum, we used the discrete version of the definition (Eq. 8):

13 $$ H(D) = \sum\limits_{{d = {D_s}}}^D {(\overline {T(d)} - {T_{{c - o}}}) * 1(\overline {T(d)} - {T_{{c - o}}})} $$

Here, *D* is day and the bar denotes the daily averaging constructed from the 6-hour ERA values, and *D*_*s*_ is the starting day of the *H* integration.

The modelled starting date of the flowering, $D_{{fs}}^{{mdl}}(s)$, for the specific station *s* was then defined as the first day when *H(D,s) ≥ H*_*fs*_. The criterion for the fitting was the RMS of the model predictions $D_{{fs}}^{{mdl}}(s)$ versus observed $D_{{fs}}^{{obs}}(s)$:

14 $$ {J_{{fs}}}({H_{{fs}}},{D_s},{T_{{c - o}}},r) = \frac{1}{{{N_r}}}\sum\limits_{{s = 1}}^{{{N_r}}} {{{(D_{{fs}}^{{mdl}}(s) - D_{{fs}}^{{obs}}(s))}^2}} \to \mathop{{\min }}\limits_{{{H_{{fs}}},{D_s},{T_{{c - o}}}}} $$

Here, *N*_*r*_ is the number of stations in the sub-region *r* and *J*_*fs*_ is the sub-regional cost function.

Fitting of *H*_*fe*_ proceeded similarly, except for the observational dataset. Since the phenological observations of the end of flowering are scarce, we used the aerobiological data from EAN. The flowering ending date for each grid cell was computed using the 97.5% criterion for the season end (Goldberg et al. 1988), after which the heat sum was computed until this date using the same start day and temperature cut-off as determined for *H*_*fs*_. Upon completion of fitting procedures for *H*_*fs*_ and for *H*_*fe*_, their difference, *ΔH*, was calculated for each sub-region.

Expectedly, the optimal fitting problem was ill-posed and the uniqueness of the solution was not guaranteed, which was a roadblock because the level of noise in the observational data was sometimes overwhelming (Siljamo et al. 2008b). Some improvements were achieved owing to the interdependence of the above parameters. As it was noticed by Linkosalo et al. (2000), *D*_*s*_ and *T*_*c-o*_ could be assumed, so that the fitting has to be done only for the temperature sum thresholds. The resulting value of the cost function *J*_*fs*_ was practically the same as for the fitting with all three parameters varying.

The assumed values for birch were *T*_*c-o*_ = 3.5°C and *D*_*s*_ = 60 (March 1). There is certainly an ambiguity in these values; one can argue, for example, that March 1 is too early in the north and may be already quite late in the south, i.e. there has to be latitude dependency of at least *D*_*s*_. In the present parameterization, however, all such trends are automatically reflected in the heat sum threshold map. Introducing any further complexity to the fitting algorithm has shown to bring no gain in terms of the final cost function value. As an additional indication of March being a good choice for pan-European starting date, Linkosalo and Lechowicz (2006) found a weak but noticeable effect of the light conditions triggering the birch leaf bud development. In such cases, the dates near spring equinox are particularly good for starting the integration because the daylight time is the same for the whole Europe.

The quality of the fitting outcome can be evaluated using several criteria. First, the residual of the fitting should be larger than the objective uncertainty of the observations: $J > \sigma_{{obs}}^2$. This requirement ensures that the model is not over-fitted to noise in the data. Second, the residual should be smaller than the sum of the observational uncertainty and the inter-annual variability of the flowering dates: $J < \sigma_{{obs}}^2 + \sigma_{{time}}^2$. This means that the model resolves this variability. Third, the large-scale features of the observed and fitted spatial patterns of the flowering dates should be similar, but the high-frequency fluctuations in the data map should be smoothed out by the model. Finally, the threshold map can be analysed using the classical work by Linsser (1867). According to Linsser’s law, for any heat-driven phenological phenomenon, a heat-sum threshold is a constant fraction of the overall accumulated effective temperature sum (ETS) over the whole growing period. In other words, the map of the heat sum threshold should be proportional to the map of ETS (the latter one can be easily computed from meteorological data over the period of active vegetation).

**Fig. 1:**
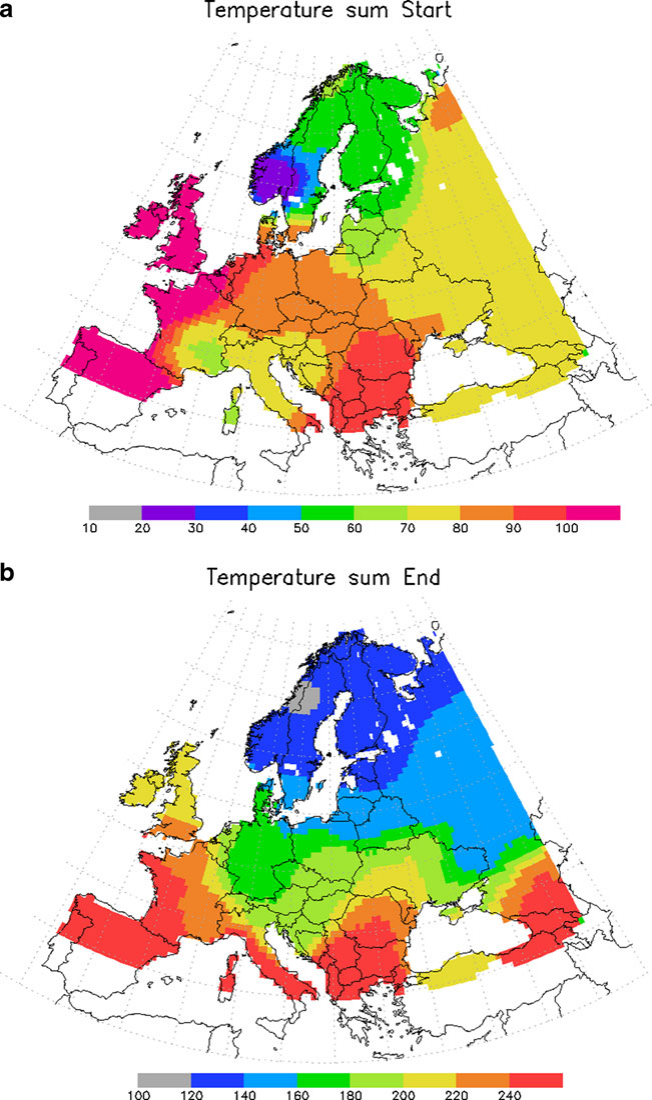
Map of the temperature sum threshold for the start of the season *H*_*fs*_ (**a**) and end of the season *H*_*fe*_ (**b**). Unit = [degree day]

**Fig. 2:**
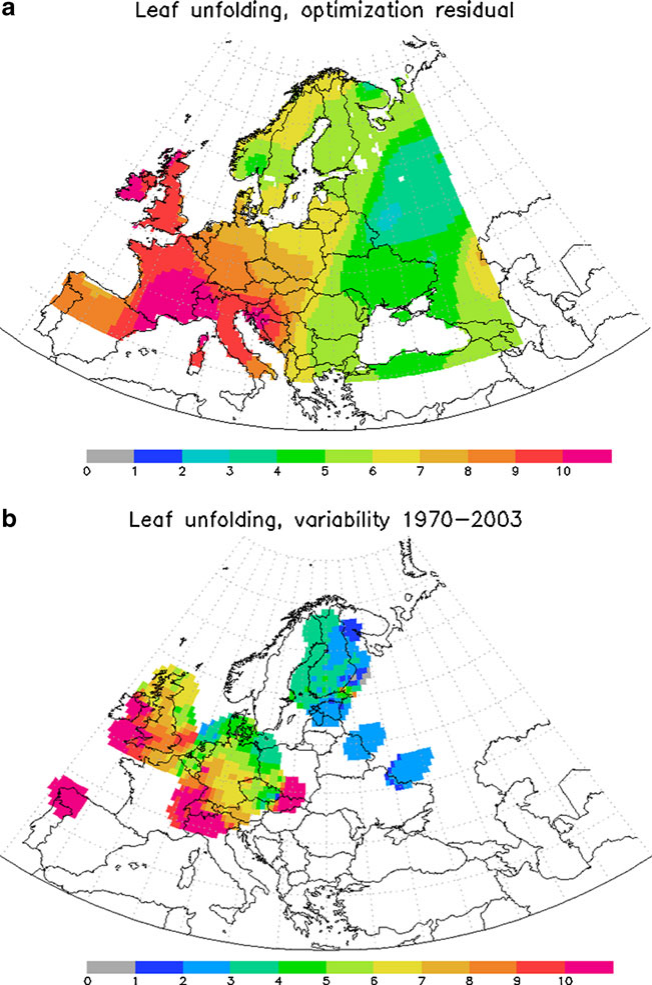
Residuals of the fitting (panel **a**) and objective irreducible uncertainties of the phenological data (panel **b**; source: Siljamo et al. 2008b). Unit = [day]

The results of the fitting (Fig. 1) satisfy all above requirements. The pattern is smooth with gradual decrease towards the north, i.e. the scatter of the input data is efficiently smoothed out. Figure 2 shows that the error of the start of flowering is indeed comparable but larger than the irreducible uncertainty in the phenological data itself, as estimated by Siljamo et al. (2008b) (these, in particular, include both natural variability of the flowering timing for different trees and the representativeness error for phenological observations). Accordingly, the procedure is conservative enough to avoid over-fitting the model to the noise in the data.

The fitting error (Fig. 2a) is lower than the inter-annual variability of the flowering dates which for some years and regions can span several weeks. For instance, in the region of the Gulf of Finland the flowering in 1999 was about 12 days earlier than the average over 1970–2003, in comparison with 6 days of the fitting RMS. Therefore, we conclude that the suggested fitting utilises (most) useful information stored in the phenological records.

**Fig. 3:**
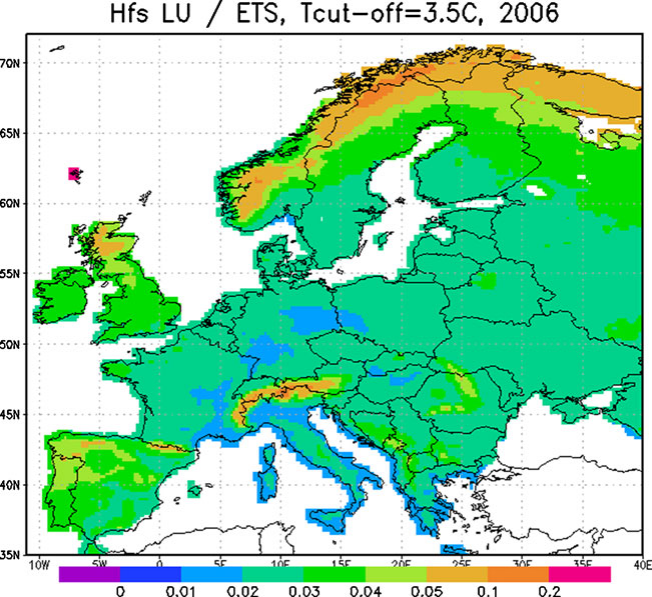
Fraction of* H*_*fs*_ in relation to ETS for 2006,* T*_*c-o*_ = 3.5°C. Relative units

The difference between the temperature sum threshold for the start of flowering *H*_*fs*_ is more than a factor of three between Southern and Northern Europe. The spatial trends of the observational uncertainty and residuals (Fig. 2) are similar, which confirms that the main contributor to the residuals is the observational uncertainty itself. Finally, the ratio of *H*_*fs*_ to ETS computed for 2006 (Fig. 3) is indeed nearly constant over the European continent. Exceptions include the mountain areas and Northern Lapland, for which the phenological information was almost non-existent and the accuracy of both Lensser’s law and our fitting is questionable. Therefore, all above mentioned quality criteria are satisfied.

Fitting of *H*_*fe*_ was less straightforward because the end of the season had to be defined from aerobiological observations which are sensitive to the long-range transport of pollen. Since observations cannot distinguish between the LRT pollen grains and those produced locally, the flowering duration tends to be overestimated. In addition, the temperature sum is accumulated throughout the whole season, including rainy and high-humidity days during which the release does not take place, which is another uncertainty pushing towards overestimation. Therefore, the *H*_*fe*_ map in Fig. 1 will require adjustments (see discussion in the companion paper). The empirical value Δ*H* = 50 degree-days has been shown to provide acceptable results.

There are some peculiarities in the threshold maps. Thus, both the start and end of season thresholds are at their highest in marine climate which can be quite warm in early spring but considerably colder during the main flowering season in April–May. An explanation of this behaviour can be the suppressed diurnal variation of temperature in the coastal regions and the much slower warming-up of the environment, which makes the cut-off temperature uncertain and probably different from the more continental conditions. As a result, the single-parameter Europe-wide fitting may not be appropriate for those regions: the cut-off temperature should possibly be taken somewhat higher than in a continental climate.

The other peculiarity is the very low thresholds in Southern (for *H*_*fs*_) and Central (for *H*_*fe*_) Norway. Despite the fact that these are coastal regions, the values there are lower than even in the Finnish Lapland. This suspicious behaviour can possibly originate from the large altitude variability in the mountainous regions and the sharp rise in the topography from the sea level up to the ridges. The ERA-40 temperature field and the fitting procedure do not resolve the narrow valleys and the quick rise of the relief. The start of the flowering of trees in the valleys is then driven by local temperatures which may have little in common with the mean temperature averaged over the 1.125° × 1.125° ERA grid cell. Also, there are too few reported observations for Norway. A similar effect shows up in the Alpine regions where the valley temperatures and the corresponding thresholds are evidently higher than up in the mountains. These regions are also outliers in the Lenssers’s ratio map in Fig. 3. To resolve these features, the grid cell should be a couple of kilometres in size, which lies outside the scope of the European model development study.

## Evaluation of the process representations in the emission module

### The main season timing and propagation model

There exist several approaches for predicting the dates of phenological phases. Our selection of the thermal type model is based on findings of Linkosalo et al. (2008), who compared the thermal time, sequential (Hänninen 1990), parallel (Cannell and Smith 1983) and flexible models (Chuine 2000; Schaber and Badeck 2003) of leaf bud burst against an independent control dataset and found that the simplest thermal time models performed better than the more complicated approaches. They suggested that the complex phenological models were over-parameterized and able to adapt to noise in the learning dataset in addition to the phenological phenomenon itself. Therefore, the simple form of Eq. 1 was considered optimal.

The second challenge was the determination of the threshold values in the Eq. 1. There are numerous parameterizations for *H*_*fs*_ (Cannell and Smith 1983; Chuine 2000; Hänninen 1990; Linkosalo et al. 2008; Menzel 1997; Rotzer and Chmielewski 2001; Schaber and Badeck 2003). However, they appeared practically incomparable with each other and applicable only to the regions and species for which they were developed; both the temperature sum threshold and the formulations for *H(t)* vary from model to model, even if their regions overlap. Attempts to generalise the formulations by introducing, for example, the latitudinal dependence of *H*_*fs*_ did not resolve the problem either. Thus, for instance, the temperature sum threshold formula with the explicit latitudinal dependence for Germany (Menzel 1997) cannot be extrapolated to the Finnish latitudes, as it would lead to negative threshold values. As a result, none of the existing thermal time model parameterizations was found directly applicable to the European-wide applications.

With no suitable model applicable for the whole of Europe, the parameters of Eq. 1 have been identified afresh, as described in the previous section. As a by-product, the identification procedure explained the differences between the existing local parameterizations; with the model parameters being interdependent, one can choose up to two of them offsetting the introduced errors with optimal selection of the remaining one(s). Since the range of reasonable choices of the assumed parameters is wide, comparison of the obtained parameterizations can be problematic. For the present model, we just unified these assumed values for *T*_*c-o*_ and *D*_*s*_.

### Short-term variations of pollen release

In this section, we analyse the short-term response of the emission module to meteorological forcing via comparison with observations. The emission fluxes are not measured explicitly, therefore the analysis has to be based on the pollen counts during the main pollen season compared with the SILAM predicted concentrations. For this evaluation, we took the EAN data for 2006 and ran SILAM with a 30 km resolution over Europe using the ECMWF meteorological data.

Such evaluation is evidently semi-qualitative and its numerical outcome should be taken with care. Particularly uncertain parts refer to temperature and relative humidity; daily observations miss the strong diurnal cycles of these parameters. The same also refers to wind, although to a somewhat lesser extent.

**Fig. 4:**
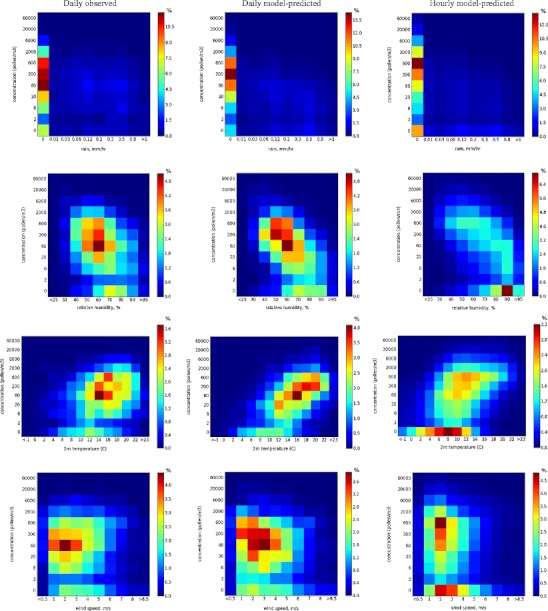
Dependence of the daily observed (*left*), daily predicted (*middle*) and hourly predicted (*right*) counts [# m^-3^] of birch pollen on the meteorological forcing: rain intensity (*first row*, mm hr^-1^), relative humidity (*second row*, %), temperature at 2 m above the surface (*third row*, °C), and wind speed at 10 m above the surface (*fourth row*, m s^-1^).* Colours* shows the fraction [%] of values falling into the corresponding range of values

In Fig. 4, the four main meteorological parameters extracted from the meteorological model fields are plotted against the observed and predicted pollen counts at aerobiological sites. As seen from the upper row, rain suppresses the pollen release, which results in a sharp decline of the predicted concentrations, in agreement with the observations. The substantial concentrations predicted and observed in a small fraction of cases should originate from the regional transport (in the model, the local emission is fully stopped for *P* > 0.5 mm hr^-1^).

Dependence on relative humidity is more complicated (Fig. 4, second row). Since the daily concentrations are plotted against humidity picked at 12:00 UTC, there are few cases with RH >80%, and the counts are indeed low during these days. A tendency of lower counts for higher RH is visible in both daily plots but the scatter is high. The diurnal variation of RH is resolved in the hourly scatter plot at the right-hand side, which reveals that the high-humidity hours (*RH* > 90%) are characterised by very low pollen counts; the intermediate-humidity levels of 90% > *RH* > 60% correspond to intermediate concentrations, whereas in most cases dry periods correspond to high counts.

The correlation of the pollen counts with temperature (Fig. 4, third row) is moderate. However, it is well seen that for the hours with high temperatures the model tends to predict higher pollen concentrations, whereas very low temperatures correspond to a low pollen load—both in predicted and observed plots.

The weakest correlation is seen for wind speed; the overall scattering is very large in both observed and predicted fields, which is the result of two competitive effects. The stronger wind speed increases the emission rate but also improves ventilation and promotes turbulent mixing. The net effect is then practically negligible, as seen from both modelled and observed plots.

The scatter plots in Fig. 4 also illustrate the mechanisms responsible for the diurnal variation of emission. Indeed, formulations (Eq. 12) do not include forcing dependence on time of the day. Birch trees do not have any sun- or light-following mechanism that would control emission of pollen (contrary to, e.g., sunflowers). Therefore, the high humidity, low temperature and low wind speed are the only factors reducing the emissions during night and causing the diurnal variation of the rates. A commonly accepted zero level of emission during the night-time can thus be reached in the model only if humidity is above the upper threshold or temperature is below the cut-off limit. In Fig. 4, such cases, whether occurring during day or night, were characterised by quite low pollen concentrations, which could still reach dozens of pollen grains per m^3^, which is in agreement with the observations.

## Comparison with another emission parameterization

In this section, we compare the formulations of the SILAM pollen emission module with the birch pollen emission parameterization developed by Helbig et al. (2004) further referred to as H04. Apart from the present model, the H04 approach seems to be the only comprehensive parameterization of pollen emission applicable at a regional-to-continental scale. It was implemented in the COSMO-ART modelling system and used for, e.g., modelling of the birch pollen episode in Switzerland in 2006 (Vogel et al. 2008). Recently, a combination of H04 with the SILAM formulations has been applied in the United States to birch and ragweed simulations (Efstathiou et al. 2011).

The principal difference between the approaches is that the H04 algorithm computes the emission flux as a product of a characteristic velocity scale with a characteristic pollen concentration, adjusted with correction functions dependent on other parameters. The flux is then driven by the turbulent stress, whereas our approach follows the temperature-driven model by Linkosalo et al. (2010). As a result, the H04 approach has to utilise the assumed duration of the flowering (included in the formulation of the probability of trees to bloom), while the current model follows the actual meteorology-driven developments.

The characteristic velocity scale in H04 is taken as friction velocity u* which is useful as a measure of the mechanically induced turbulence near the surface. However, it may not be the ideal parameter describing the mechanical stress to the birch flowers, which are several meters from the surface and located in the tree crown where the similarity theory is not applicable. At this height, the stress could be represented by regular wind blow through the canopy rather than turbulence. The closest standard meteorological variable would be the wind speed at 10 metres above the displacement height (in other words, near the tree top), which is the variable used in SILAM. It is a diagnostic variable in all meteorological models and incorporates indirectly the horizontal wind shear stress, thus making the involvement of u* unnecessary.

The turbulence-driven stress would become dominant in free convection conditions, in other words, in unstable stratification and low mean wind conditions. The corresponding term is absent from H04, whereas the present approach includes it via the convective velocity scale w*.

The impacts of precipitation and relative humidity to the emission rate are similar in both parameterizations. Temperature is also an emission promoter in H04 formulations; however, it does not play as central a role as in our model.

One of the key factors in H04 is the leaf area index (LAI), an increase which reduces the flowering intensity. We did not include this parameter at all because its relation to the birch flowering stage is neither straightforward nor easy to determine. The usual presumption that the flowering finishes before the leaves reach full size (in order not to inhibit the pollen dispersal) corroborates the H04 approach. Nevertheless, in the dataset (Siljamo et al. 2008b) the bud burst day was often before the first flowering day. As a result, LAI might start rising already before the flowering begins. However, LAI can still be useful in the case of prolonged flowering in wet, cold weather when leaves grow up before all pollen grains are released.

Another process not included in the current parameterization but considered in H04 is pollen resuspension. As noted by H04, this process is very poorly known and can only take place when the wind is very strong and gusty (above 15 m sec^-1^). Its influence can possibly be noticed at the end of the season, in flat terrains and in dry conditions only. Therefore, we considered it as a rare phenomenon, the uncertainty of which is larger than the potential gain of its inclusion into the model.

## Summary

The suggested pollen emission model for birch follows the concept of thermal time phenological models and, in particular, the double-threshold temperature sum approach determining the development of flowering during the whole spring season.

Apart from temperature, pollen release rate is modulated by ambient humidity, precipitation and wind speed. Higher humidity and rain suppress the release, whereas stronger wind promotes it. Atmospheric turbulence is taken into account via the turbulent velocity scale and thus becomes important only in cases of near-free convection.

The probability of an individual tree to enter the flowering stage is considered via the uncertainty of the temperature sum threshold determining the start of flowering.

The end of the season is described via the open pocket principle, according to which flowering continues until the initially available amount of pollen is released.

Numerical values of the model parameters—temperature sum threshold for the start and end of flowering, critical levels of relative humidity and precipitation intensity, and characteristic wind speed—were identified via optimal fitting to the European phenological and aerobiological data.

The model does not include the explicit diurnal variation forcing, which is obtained as a by-product of the meteorological forcing. Pollen resuspension and the relation between flowering and leaf area index are also left out of the parameterization due to high uncertainties in both parameters and their unclear relation to the pollen release processes.

The model processes have been qualitatively evaluated using the observed pollen counts in 2006, which were related to the meteorological input data and compared with the model predictions. The quantitative analysis of the model performance is the subject of the companion paper.

The described model is freely available from the SILAM model website at http://silam.fmi.fi.
